# Clinical Translational Potential in Skin Wound Regeneration for Adipose-Derived, Blood-Derived, and Cellulose Materials: Cells, Exosomes, and Hydrogels

**DOI:** 10.3390/biom10101373

**Published:** 2020-09-27

**Authors:** Trivia Frazier, Andrea Alarcon, Xiying Wu, Omair A. Mohiuddin, Jessica M. Motherwell, Anders H. Carlsson, Robert J. Christy, Judson V. Edwards, Robert T. Mackin, Nicolette Prevost, Elena Gloster, Qiang Zhang, Guangdi Wang, Daniel J. Hayes, Jeffrey M. Gimble

**Affiliations:** 1 Obatala Sciences Inc., New Orleans, LA 70148, USA; andrea.alarcon@obatalasciences.com (A.A.); xiying.wu@obatalasciences.com (X.W.); 2 LaCell LLC, New Orleans, LA 70148, USA; 3 Panjwani Center for Molecular Medicine and Drug Research, International Center for Chemical and Biological Science, University of Karachi, Karachi 75270, Pakistan; omohiudd@tulane.edu; 4 Walter Reed National Military Medical Center, Bethesda, MD 29814, USA; jmmotherwell@gmail.com; 5 United States Army Institute of Surgical Research, JBSA Fort Sam Houston, San Antonio, TX 78234, USA; anders.h.carlsson.ctr@mail.mil (A.H.C.); robert.j.christy12.civ@mail.mil (R.J.C.); 6 Southern Regional Research Center-USDA-ARS, New Orleans, LA 70124, USA; vince.edwards@usda.gov (J.V.E.); Robert.Mackin@usda.gov (R.T.M.); nicolette.prevost@usda.gov (N.P.); 7 Department of Chemistry, Xavier University of Louisiana, New Orleans, LA 70125, USA; egloster@xula.edu (E.G.); qzhang@xula.edu (Q.Z.); gwang@xula.edu (G.W.); 8 Department of Biomedical Engineering, State College, Pennsylvania State University, Centre County, PA 16802, USA; djh195@psu.edu

**Keywords:** adipose-derived stromal/stem cells (ASC), blood, burns, cellulose, exosome, platelets, pressure injury, pressure ulcer, secretome

## Abstract

Acute and chronic skin wounds due to burns, pressure injuries, and trauma represent a substantial challenge to healthcare delivery with particular impacts on geriatric, paraplegic, and quadriplegic demographics worldwide. Nevertheless, the current standard of care relies extensively on preventive measures to mitigate pressure injury, surgical debridement, skin flap procedures, and negative pressure wound vacuum measures. This article highlights the potential of adipose-, blood-, and cellulose-derived products (cells, decellularized matrices and scaffolds, and exosome and secretome factors) as a means to address this unmet medical need. The current status of this research area is evaluated and discussed in the context of promising avenues for future discovery.

## 1. Introduction—Skin Wounds as a Medical Challenge

The skin as an organ is uniquely exposed to the environment and is responsible for maintaining the integrity of the body’s fluid dynamics and immune system. Thus, any breach through the skin’s barrier has the potential for severe morbidity and mortality consequences. In civilian and military patient populations, life-threatening skin wounds result from burns and thermal injuries, from trauma secondary to cuts, gunshots, high energy blasts, and vehicular accidents, or from decubitus ulcers involving ischemia and reperfusion injury. Skin thermal injuries account for 5% to 15% of combat casualties and 45% of all infections; the military anticipates that burns will present a substantial unmet medical need in future conflicts [1,2,3,4,5,6]. In the U.S., nearly 2.5 million individuals experience burns annually. Of these, 30,000 will be severe enough to require hospitalization in a burn unit specialty treatment center. Similarly, it is estimated that over 10% of U.S. nursing home residents over the age of 70 will experience a pressure ulcer or pressure injury annually [7]. Between 2 and 4% of the healthcare economies of most advanced industrialized countries is expended in the treatment of pressure injuries and their complications [8,9]. Skin wounds can be infected by bacterial or fungal contaminants, leading to patient outcomes being complicated by acute and recurrent bacteremia, impaired wound repair, osteomyelitis, sepsis, and death [2,3,4,5].

There have been considerable advancements in the understanding of the biochemical pathways and mechanisms underlying skin regenerative therapies, leading to explorations into the use of cells and cell-derived cytokines and exosomes, as well as hydrogels (recently reviewed in [10,11,12,13,14,15,16,17,18,19]) (Table 1). While decellularized dermal scaffolds are routinely employed [20], only a PDGF (Platelet Derived Growth Factor)-related product has received regulatory approval among the cytokines, while no cell therapy has widespread approval in the U.S. [21,22]. Thus, rather than reliance on sophisticated biologics, the current standard of care for skin wounds continues to employ preventive measures and surgical interventions involving debridement, autologous skin grafts, or the introduction of decellularized allogeneic or xenogeneic biological scaffolds. These approaches, based on decades of experience, are well established and validated with respect to outcomes [23,24]. Furthermore, the integration of negative pressure wound vacuums and hyperbaric oxygen has improved outcomes with these surgical therapies [25].

Thus, an opportunity remains to develop alternative approaches and products to accelerate and enhance skin wound regeneration. Optimally, such a product(s) would display one or more of the following features:

(1) Stable under room temperature storage;
(2) Suitable for topical or injectable delivery;
(3) Antimicrobial, analgesic, or hemostatic properties;
(4) Accelerates wound repair by promoting conductive or inductive regenerative mechanisms.

This review article delves into the potential application of existing and evolving adipose-, blood-, and cellulose-derived biological products in the context of skin wound healing and regeneration, while highlighting avenues for future investigation.

## 2. Adipose-Derived Cells

**Figure 1:**
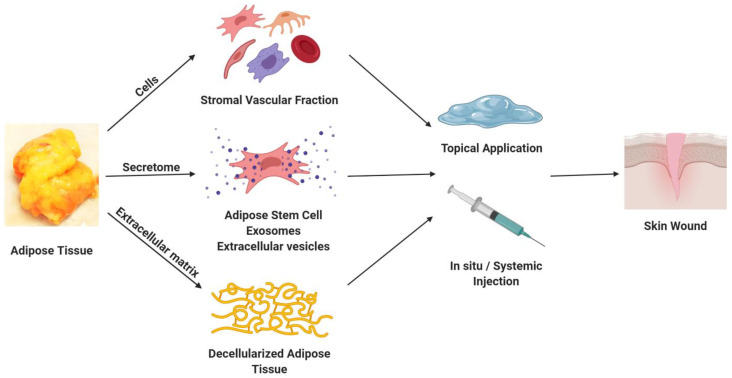
The processing and isolation of cells and a decellularized extracellular matrix (ECM) scaffold from adipose tissue and their application for the repair and regeneration of skin wounds.

Over two decades ago, adipose tissue was identified as a rich and abundant source of regenerative cells. In pioneering work by Zuk, Katz, and their colleagues at the University of Pittsburgh and the University of California Los Angeles, the stromal vascular fraction (SVF) cells isolated by collagenase digestion from subcutaneous adipose tissue of patients undergoing elective liposuction were found to contain an adherent population of adipose-derived stromal/stem cells (ASC) [32,33]. Following culture expansion, these ASC (originally termed processed lipoaspirate or PLA cells) displayed adipogenic, chondrogenic, myogenic, and osteogenic differentiation in vitro [32,34]. The multiple lineage potentiality of the ASCs was an indicator of their utility as a regenerative cell therapy capable of repairing or replacing damaged or injured tissues such as skin. The SVF cells and ASC have been further characterized based on the presence or absence of discrete surface antigens, including CD13, CD31, CD34, CD44, CD45, CD73, CD90, and CD105 [33,35,36]. Further studies have demonstrated the ability of ASCs to modulate mixed lymphocyte reactions in vitro and to display immunosuppressive properties in vivo [37,38]. Through the use of enzymatic isolation methods, independent laboratories have advanced SVF cell and ASC production as current good manufacturing practices (cGMP) to create clinical-grade cell products that are suitable for the treatment of skin wounds, soft tissue cosmesis, or bone defects [39,40,41,42]. These approaches have relied on the use of the patient’s autologous serum or the use of human platelet lysates for cell culture and expansion [43,44]. Moreover, in related work, a number of companies, including Cytori, GID (Get It Done), and Tissue Genesis, have reported the development of closed-system devices to enzymatically process and isolate SVF cells within the operating room [45,46]. Studies in preclinical rodent models have begun to examine the SVF cells and ASCs isolated with conventional laboratory methods or closed-system devices in the treatment of pressure injuries and related skin wounds [11,47,48]. These preclinical “proof of principle” analyses have determined that both freshly isolated and cryopreserved human ASCs can accelerate and enhance the rate of skin wound healing [48] (Figure 1). Indeed, by performing the isolation procedure on lipoaspirates harvested at the time of a single operation and immediately implanting the recovered cells, regulators have authorized such autologous adipose cell products to advance into clinical trials [49]; however, to date, none of these devices are used routinely in the United States as a basis for skin wound therapy. Furthermore, while sophisticated adipose-derived cell therapies are feasible as elective procedures in a civilian hospital of an industrialized country, these would not be practical in less advanced environments, such as a poorly resourced hospital in a developing nation or under conditions encountered on the battlefield. Thus, there is a critical need for alternative adipose-derived products that retain regenerative properties found in cells. Moreover, eliminating the liability associated with complex isolation processes and cryopreservation remains a universal goal for product development.

## 3. Adipose-Derived Secretomes, Exosomes, and Microvascular Tissues

The regenerative properties of SVF cells and ASCs have been attributed, in part, to their paracrine or exocrine actions [50,51,52]. Dependent on their exposure to inductive agents such ligands binding to the surface toll-like receptors (endotoxin, poly dI/dC), ASCs are activated to condition their culture medium with anti- or proinflammatory cytokines, as well as angiogenic and vasculogenic growth factors [53]. These proteins can be secreted directly into the medium or are endocytosed and enclosed within extracellular vesicles such as exosomes. The exosomes are membrane-bound microvesicles that are characteristically enclosed by membranes containing tetraspanin proteins (CD9, CD63, CD81), lysosomal-associated membrane proteins (LAMP1/2), and tumor susceptibility gene 101 (TSG101) as hallmarks [54,55]. Exosome or extracellular vesicles derived from human ASCs have been used to accelerate the healing of full-thickness skin wounds in rodent models [54,56]. Following injection into the wound, the ASC extracellular vesicles activated the AKT and ERK pathways to improve closure, collagen deposition, and vascularization [56]. In addition to storing proteins, exosomes and microvesicles serve as intra-cellular delivery vehicles for microRNAs. The ASC-derived microRNAs are capable of modulating wound healing by driving lineage differentiation along the adipocyte, chondrocyte, myocyte, and osteoblast pathways [57]. Additionally, it is hypothesized that exosomal microRNAs similarly modulate pathways involved in the inflammatory, proliferative, angiogenic, and remodeling phases of wound healing [54]. A number of methods are used to isolate and concentrate exosomes, including differential centrifugation, ultra-centrifugation, size exclusion chromatography, immunoselection, and precipitation [55]. To maximize recovery, mesenchymal stromal cells (MSC), including ASCs, are cultured on microfiber or microcarrier bioreactors to achieve the highest possible density of viable cells [55]. The ASCs or MSCs must be cultured using media components that are compatible with cGMP and regulatory approval, i.e., endotoxin-, mycoplasm-, xenoprotein-, and viral-contaminant free reagents. Lyophilized SVF cell or adipose microvascular tissue extracts serve as an alternative to direct exosome purification [58]. After reconstitution and injection or topical application, these products accelerated healing in a murine pressure injury wound model through mechanisms involving the induction of vasculogenic cytokines [58]. Multiple academic laboratories and biotechnology companies are pursuing ASC-derived exosome products as potential therapies for skin wound lesions and other disease conditions [56,59]. When lyophilized or freeze-dried, these products have the potential to remain stable for extended periods of storage at room temperature, making their delivery feasible under circumstances where refrigeration is unavailable. While exosome products will need to be evaluated in randomized, controlled, and preferably blinded clinical trials, recent studies have demonstrated preliminary evidence of safety and efficacy in patients infected with SARS-CoV-2 who were administered bone marrow MSC-derived exosomes to improve oxygenation, reconstitute immunity, and downregulate the inflammatory cytokines associated with the “cytokine storm” of COVID-19 [60]. Thus, it is likely that ASC-derived exosomes will display a comparable level of safety and efficacy based on the observed similar modes of action [57].

## 4. Extracellular Matrix (ECM) from Decellularized Adipose Tissue

The methodology for decellularizing adipose tissue was pioneered by Flynn and colleagues [61,62,63,64,65,66,67,68,69,70,71,72,73] and has since been confirmed by multiple independent laboratories [74,75,76,77,78,79,80,81,82]. These methods combine biological (enzyme digestion), chemical, and physical processing steps to achieve the uniform and consistent manufacture of protein scaffolds depleted of contaminating genomic DNA and lipids. Obatala scientists, in collaboration with partners at Pennsylvania State University (PSU), Tulane University, and Western Ontario University, have developed and published on decellularized human adipose tissue-derived extracellular matrix (ECM) hydrogel, commercially termed “AdipoGel™” [70,83,84,85,86]. This hydrogel or scaffold is prepared using a combination of chemical and mechanical processing followed by protease digestion as a final modification prior to sterilization. In vitro, AdipoGel is capable of supporting adipose-derived stromal/stem cell (ASC) proliferation and adipogenic and osteogenic differentiation; in vivo, AdipoGel promotes critical-sized endochondral bone defect repair [83,84,85]. Furthermore, the decellularized adipose-derived scaffold can be chemically modified with thiol methacrylate and cross-linked to create a cytocompatible matrix that can be “tuned” to achieve biomechanical properties appropriate for the promotion of either soft or hard tissue regeneration [87]. Mass spectrometry analyses demonstrate that the AdipoGel proteome is enriched for ECM proteins, including collagens, fibrillin, laminin, nidogen, and other adhesion molecules, while the AdipoGel proteome is depleted in cytoplasmic and nuclear protein contents relative to native tissue [70,86]. The decellularized tissue can be prepared as a lyophilized scaffold or as a liquid hydrogel. Both forms are compatible with in vivo applications, including subcutaneous implantation, injection for soft tissue regeneration, or as a sheet for use in orthopedic repair procedures [70,83]. Likewise, independent studies have demonstrated that their decellularized adipose tissue scaffolds can be used to create adipose depots when implanted into nude mice [82]. Furthermore, the scaffolds were maintained safely for up to 4 months without evidence of complications when implanted subcutaneously under the dorsal skin of the wrist in human subjects, with a single patient showing safety and efficacy after 16 months of implantation [82]. In an elegant randomized follow-up study, Kokai et al. (2020) implanted their adipose-derived ECM product by injection into the pannus of patients scheduled for elective abdominoplasty [88]. Subsequent analysis of the resected tissues after 3 or 6 months demonstrated safety and efficacy, with histological evidence of remodeling at 3 months and adipogenic differentiation of host ASCs by the 6-month time point [88]. These analyses lay the foundation for regulatory approval of the decellularized adipose tissue products for soft tissue regeneration. It remains to be determined if the product will be categorized by the FDA as a tissue, device, or biological material. Regardless of the regulatory division, the product will require additional safety and efficacy testing in clinical trials, with post-market review over a period of years to monitor subjects for any unanticipated adverse events. Currently, the Renuva™ decellularized adipose allograft matrix product is available from the Musculoskeletal Transplant Foundation, and this has been optimized in pre-clinical animal studies (MTF Biologics, Edison, NJ, USA) [89].

## 5. Blood Products

**Figure 2:**
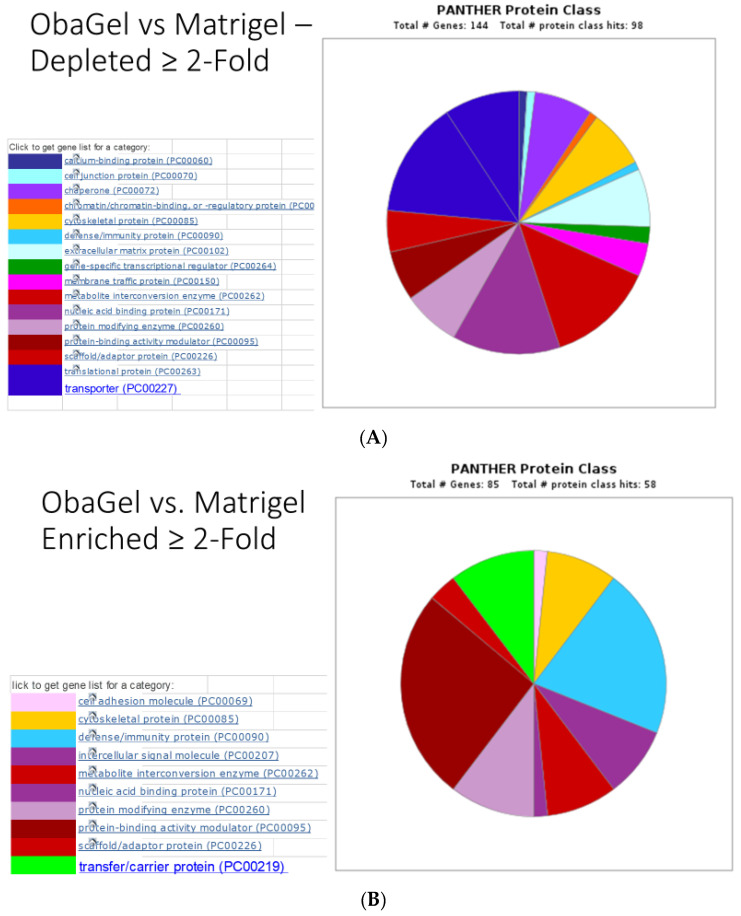
PANTHER pathway analysis of ObaGel vs. Matrigel proteomes based on (**A**) >2-fold-enriched and (**B**) >2-fold-depleted proteins. The relative abundance of proteins in representative ObaGel and Matrigel lots was determined using TMT (Tandem Mass Tag) isobaric mass tagging using tandem mass spectrometry. Those proteins enriched > 2-fold (**A**) or depleted > 2-fold (**B**) between ObaGel and Matrigel were evaluated using PANTHER pathway analyses and plotted using a pie chart.

Investigators at the U.S. Army Institute of Surgical Research have used polyethylene glycol (PEG) modification chemistry to adapt fibrin hydrogels prepared from purified fibrin and thrombin or from platelet-rich plasma as therapeutics for burns and full-thickness skin wound models [90,91,92,93,94,95]. These materials can be applied topically following creation of the lesion to enhance healing. The platelet-rich, plasma-derived hydrogels are effective scaffolds for delivery of ASCs, promoting their angiogenic properties in vitro and in vivo [95]. Additionally, the scaffolds can be employed to deliver antimicrobial silver nanoparticles or ASCs to infected burns, with significant reductions in bacterial colony counts and proinflammatory cytokine expression [90]. In similar studies, Obatala has developed and commercialized a proprietary, patent pending hydrogel derived from human blood (ObaGel™) (USPTO (United States Patent and Trademark Office) Application *“Biological Scaffolds, Products Containing Biological Scaffolds and Methods of Using the Same” #20200078411, March 20, 2020*). Published studies have demonstrated that ObaGel supports the growth and differentiation of adipose-derived cells along the adipogenic and endothelial lineages [96]. Unpublished studies have demonstrated that ObaGel supports robust angiogenesis and vasculogenesis in vivo. While the proteome of AdipoGel has been described based on an unbiased global mass spectrometry analysis, no such comparable data has been published previously with respect to ObaGel. To address this, a pilot study has quantitatively compared the ObaGel proteome to that of Matrigel™, an extracellular matrix protein derived from the Englebreth–Holm–Swarm murine tumor, which is known to be enriched in basement membrane proteins, including collagen IV, entactin, and laminin [97]. The direct comparison using the PANTHER (Protein Analysis Through Evolutionary Relationships) bioinformatic software program [98,99] between the ObaGel and Matrigel proteomes demonstrates that ObaGel is enriched by 2-fold or more in proteins related to the coagulation and prothrombin activation pathways, as well as those involved in integrin, endothelin, and Wnt signaling. In contrast, Matrigel is enriched by 2-fold or more in proteins related to the cytoskeletal Rho-GTPase regulation, inflammation, and integrin signaling; included among these are laminin, vimentin, and nidogen, all of which are >5-fold enriched (Figure 2, Supplementary Methods, and Supplementary Excel). While both Matrigel and ObaGel undergo gelling when converted from 4 °C to 37 °C, the gelling of ObaGel can be accelerated by the presence of ASCs. Currently, ObaGel is generated as a research-grade product from raw goods sourced from screened human donors enrolled at blood centers certified by the American Association of Blood Banks (AABB). As such, it is likely that ObaGel can be readily and rapidly converted from an investigative tool into a cGMP product eligible for FDA (Food and Drug Administration) regulatory approval and subsequent clinical application.

## 6. Modified Cellulose Products

Cellulose applied in its many derivatized forms and structures has made major inroads in the last two decades as a useful biopolymer in both wound healing and tissue engineering, and interest in its uses has paralleled other relevant biopolymers applied functionally in dressings and implantables, including collagen, alginate, chitosan, and hyarulronan. Cellulose, a biological polymer synthesized by bacteria and plants, has been used as a wound dressing in the form of bandages and gauze coverings for centuries [100]. Cellulose materials possess biophysical properties consistent with skin wound healing, including antimicrobial, hemostatic, hydrophilic, immune barrier, and mechanical or structural features, making them ideal as skin-contacting materials. It is beyond the scope of this review to encompass the worldwide effort in biomedical sciences endeavoruing to apply cellulosic and nanocellulosic materials to implantables and wound dressings. Numerous recent reviews have given an in depth treatment in this regard [101,102,103,104]. However, relevant to stem cells and extracellular matrix protein science, as outlined above, it is important to consider some of the structural modifcations and functional value that cellulose’s use has been found to impart to wound healing and tissue engineering approaches. Thus, we will consider some of the relvant issues that have been addressed in its use to promote healing in chronic wounds and as a biocompatible scaffold to support tissue engineering approaches.

The intense interest in using cellulose and nanocellulose for wound healing and tissue engineering in recent years is due to their non-immunogenic and biocompatible properties, which are attributable in part to their low propensity to absorb proteins, making cellulose and nanocellulose compliant in biological milieus [102,103,104,105,106]. Compared to proteins, cellulose polymers are relatively non-immunogenic [107,108]. In addition, it has been shown that albumin, which is the protein of highest concentration in wounds, does not unfold on cellulosic materials [109,110,111]. However, the human body lacks enzymes necessary to completely breakdown of cellulose materials, however through chemical modification it is possible to augment the biodegradation [103]. Although cellulose scaffolds are relatively inert, once introduced the long-term impact of residual cellulose materials in situ remains to be determined [112]. Thus, it has been suggested that in addition to the existing physicochemical and cytotoxicity characterization assays routinely performed on cellulosics for biomaterial use [113,114,115], an unbiased global characterization of the cellulose scaffold proteome using mass spectrometry approaches is warranted.

## 7. Cellulose in Wound Healing

“Intelligent’’ dressings may be defined as materials that respond to specific changes in the wound environment (i.e., exudate volume) by altering the structure or properties to bring about a useful result (i.e., moist wound-healing conditions) [116]. The term was introduced a quarter-century ago in the context of a porous polyurethane material with a self-adjusting water vapor rate of transmission properties [117]. Since then, a variety of favorable wound healing conditions and pathologies have been targeted in dressing designs that either detect or modulate wound conditions. For example, wound temperature, pH, bioburden, and moisture levels of the wound are measurable indicators of pathology [118,119]. Some of these approaches interface to point-of-care treatment and in situ promotion of wound healing.

**Figure 3:**
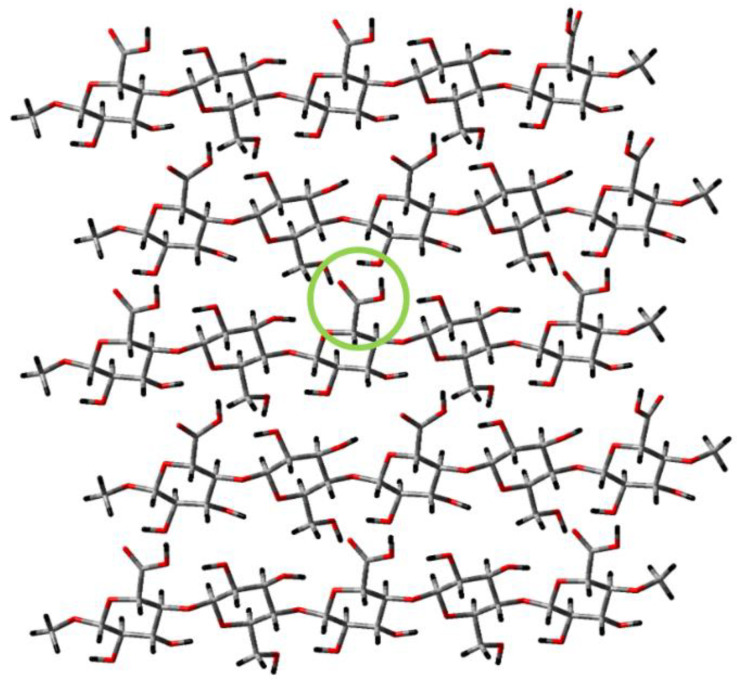
Computational model depicting the TEMPO-oxidized cellulose surface, featuring five parallel cellulose strands capped by methyl groups on each end. The geometry of the layer was optimized using Gaussian computational chemsitry software via semi-empirical PM3 calculation, and the colors of the atoms correspond as such—grey for carbon, red for oxygen, and black for hydrogen. The green ring highlights one of the carboxylate groups on the surface generated during the TEMPO oxidation process. This functional group is used as a chemical modification point for the nanocellulose structures.

The value of cellulose in intelligent dressing design is its ability to be modified in a variety chemical and physical forms. This is due to its pendant anhydroglucose hydroxyls and hydrogen bonding network, which can vary depending on the source or modification (Figure 3 depicts a model of TEMPO-oxidized cellulose). Cellulose and its derivatives have been classically identified based on their X-ray crystallography structures, which have been characterized as cellulose I–IV [120,121,122,123,124]. However, most cellulose-containing biomaterials, including dressings and tissue engineering scaffolds, are generally cellulose I, which denotes that in the crystalline state cellulose chains are parallel in the unit cell (the smallest possible volume of a cellulose crystal). Cellulose II examples abound in biomaterials where regenerated cellulose is employed in dressings and implantables, and the difference in structure is characterized as an antiparallel orientation of the cellulose chains arising when cellulose is placed in solution to make regenerated cellulose, e.g., often employed in oral surgery. Cellulose I is present in bacterial cellulose (BC), which has received considerable attention. At the microfibril level, structural variations in BC are observed. These result from the bacterial synthesis itself, which tends to create a gelatinous film in its natural state, which assumes a three-dimensional network compatible with the ECM [125]. BC has been investigated intensely for its wound healing and artificial tissue applications for the last two decades in implantables and extracorporeals [126,127,128].

Bacterial nanocellulose (BNC), a 3D interconnected network of nanofibrils, is similar to collagen networks [129] and can hold up to 99% water in its native state [130]. It has high mechanical strength, material purity, and high microporosity in the wet state [131]. These exceptional material properties make BNC a novel biomaterial for many potential medical and tissue engineering applications [132]. Recently, BNC with a cellulose content of 15% or higher has been proposed as an auricular cartilage replacement implant material due to its similarities with the mechanical requirements of the human body. Additionally, the biocompatibility of this BNC with increased cellulose content was investigated to evaluate its response in vitro and in vivo [126]. These experiments were carried out using cylindrical BNC structures (Ø48 × 20 mm), which were produced, purified in a built-in house perfusion system, and compressed to increase the cellulose content in the hydrogels.

Other uses of BNC for wound healing have illustrated that cellulose scaffolds can be formed into tubes and developed as a blood vessel implants to treat cardiovascular diseases. These tubes are composed of an inner diameter of 6 mm or less and are upwards of 25 cm in length, with varying material properties dependent upon the method of preparation. The implants placed in animals show good biocompatibility and were well integrated into the host, both in the inner and outer surfaces, a year after implantation [133]. Results such as this provide good evidence for the long-term use of BNC implants for wound repair and regeneration in the human body.

Cellulose materials have been used to provide localized delivery in a time-dependent, controlled-release manner for drugs. Molecules such as antimicrobial silver nanoparticles, minocycline, and octenidine have been incorporated into cellulose-based dressings to produce an antibacterial environment over weeks of wound recovery [134,135,136,137]. Composites made from BNC and collagen have been shown to be antioxidant, while BNC films containing silver chloride have been produced as antibacterial dressings [133]. Changes in drug diffusion rates are accomplished by manipulating the nanoparticle surface area to volume ratios and incorporation of additional hydrogels with distinct physicochemical properties [103].

It is important to note that the efficacy of ECM and growth factor-based dressings and gels applied to the chronic and burn wound environment can be subject to breakdown due to the high protease titer of the non-healing wound unless detected and modulated to acute wound levels [138]. An approach to modulating the protease titer in the chronic wound was first reported in 1999 with cellulose-based dressings formulated with inhibitors [139]. In subsequent decades a variety of protease modulating dressing paradigms have been investigated and marketed [140,141]. An example of synthetically modified dressings that impact wound healing can be found in some of the first protease modulating dressing approaches reported where derivatives of cellulose and its use as a carrier of protease inhibitors were identified for their ability to neutralize the high protease titer that can break down growth factors and ECM in chronic wounds [140]. This work advanced to the demonstration of oleic acid to albumin transfer as a form of protease inhibition. Protease modulation in the chronic wound is critical to lowering harmful protease levels that inhibit the efficacy of growth factor and ECM adjuvants for wound healing as described in (Table 1). For example, Regranex contains carboxymethylcellulose which is negatively charged and binds positively-charged proteases as elastase. It is also noteworthy that the interface of nanocellulose-based protease sensors with protease-modulating dressings has been shown to be an important step toward detecting proteases by way of point-of-care diagnostics [142] and enhance the efficacy of biopharmaceutical approaches to wound care by.by improving the wound healing trajectory in chronic wound patients.

## 8. Cellulose Tissue Engineering Applications

Another example of a cellulose modification with promise toward response to the wound and implantation environment can be found in work where cellulose was selectively modified through a methylation reaction to give rise to a “smart hydrogel”—named as such because the properties can be tuned via external forces, such as changes in temperature and pH [143]. For example, at 4 °C a methylcellulose analog dissolved in water is hydrophilic and maintains an aqueous state; however, when the methylcellulose exceeds it low critical solution temperature (LCST) it becomes hydrophobic and forms a hydrogel (known as the “sol–gel” transition). This thermo-responsive behavior has been shown to be completely reversible when the external force is removed as well. These hydrogel characteristics can be further tuned based on the concentration, degree of methylation, and ambient salt concentration, such that the LCST can be modified for physiological conditions producing hydrogels better suited for human implantation [144].

Modifications have also been employed to introduce unique binding sites, such as classic RGD (Arginine-Glycine-Aspartate) receptor ligands for integrins incorporated into the cellulose scaffolds [145]. Furthermore, the chemical modification of cellulose allows the scaffold to be fine-tuned to display organ- or tissue-specific features with respect to the elasticity, compressive and mechanical strength, and viscosity. For instance, modifying how polymers adhere to the cellulose surface can alter the material’s thermal properties and tensile strength, producing biomimetic bone structures for bone engineering and repair. Cellulose-based scaffolds mixed with hydroxyapatite have been used to mimic the ECM of natural bone, which allows for the proliferation and differentiation of mesenchymal stem cells towards osteoblasts [146,147].

## 9. TEMPO-Oxidized Nanocellulose

TEMPO-oxidized (TO) nanocellulose (see structure in Figure 3) tends to assume a hydrogel-like state due to high moisture absorption, especially compared with other forms of nanocellulose. For example, we have observed that TO-nanofibrillated cellulose swells several orders of magnitude more than nanocrystalline forms. Hydrogels of TO cellulose nanocrystals (CNCs) are typically composed of complex hydrophilic polymers that absorb a high water content (see Figure 3). Work on combining TO-CNCs with ECM to promote wound healing is a fertile area [148,149]. TO cellulose nanofibers (TO-CNFs) have been the attention of considerable interest for innovative wound healing and development of tissue engineering materials in recent years. This in part has been due to an improved understanding of the properties of TO-CNFs that are conducive to its application in moist wound healing, chronic wound modalities, and tissue scaffolding. For example, their application in moist wound healing has been elucidated, as well as their antimicrobial activity, based on the inhibition of P. aeruginosa found in burns and chronic wounds [150]. Subsequently, Jack et.al. [151] examined the effect of hydrogel dispersion on the growth of *Pseudomonas aeruginosa* (PAO1) in a suspension. Its surface and bulk structure (aerogels and films) were observed to influence biofilm formation, and changing the porosity of the material had no effect on pseudomonal virulence factor production. Additionally, TO-CNFs exhibited no toxicity to 3T3 cells and primary human skin cells.

Some nanofibrillar cellulose biomaterials have advanced to clinical trials for burn treatment and skin regeneration [152,153]. These must strike a delicate balance between adherence to the wound bed with promotion of re-epithelialization and with subsequent dressing removal without damage to the newly regenerated skin [103,152,153]. The ability of a dressing to encourage wound healing and repair relies heavily on the physical properties of the dressing, such as its fluid uptake and capacity to remain hydrated, the flexibility of the bandage, and the porosity. All of these features can be modified by varying the chemical composition of the dressing, allowing for tuning of a dressing for a specific wound [154,155]. Features such as porosity have proven to have particular importance [156,157,158] and can be tuned through the inclusion of cellulose nanowhiskers into the scaffold [159]. While high porosity enhances regeneration by allowing cell migration and invasion within the cellulose scaffold, low porosity is associated with reduced bacterial infection and water loss in the presence of a compromised skin barrier [160].

In the field of stem cell therapies, the efficiency of these therapies often relies on the cells being adequately delivered to and retained by the target location. Choosing the correct cell carrier—one which the stems cells readily adhere to and will remain within the wound—is of vital importance for healing [161]. Recently, CNFs have been utilized as carriers for stem cells, and have been shown to improve cell retention at the wound site and prevent the cells from being moved by the bloodstream. Compared to CNCs, CNFs are more flexible and are often only of nanometer thickness in diameter, yet microns in length, providing a significant surface-area-to-volume ratio [161,162]. In addition, the substantial number of hydrogen bonding groups on the surface provide the capacity for cross-linking and entanglement to strengthen the material. Stem cells seeded onto the CNF show elongated shapes and cytoplasmic extension of the cells, illustrating that these carriers encourage the proliferation of stem cells [161,162]. Additionally, the CNFs were determined to be non-cytotoxic to the stem cells, and the thread-supported growth reached culture confluence within a week, which allows for a patient’s own adipose tissue to be used [161].

While two-dimensional surfaces do not adequately mimic the in vitro environment, it has been shown that CNF can be mixed into a matrix and can form hydrogels to be used as three-dimensional scaffolds for stem cell implantation. Similarly to the other forms of cellulose, these hydrogels have tunable physical and chemical properties and have demonstrated a lack of cytotoxicity [163]. Previous studies have shown these hydrogels can be used to promote the formation of human liver cells into a 3D spheroid [164]. While cellulose cannot be naturally broken down in the human body, the CNF hydrogels can be removed using cellulase, an enzyme which breaks down cellulose into non-toxic sugars without affecting animal cells. The enzymatic breakdown removes the CNF hydrogel while preserving the 3D structure of the stem cell spheroids and does not hinder the pluripotency of the cells [163]. These 3D spheroids are then poised for directed differentiation into tissues for wound repair and regeneration.

Despite these potential limitations, cellulose and nanocellulose materials present distinctive and potentially advantageous properties with respect to skin wound healing relative to adipose and blood-derived scaffolds. Indeed, the opportunity exists to create combinations using these three biomaterials in varying ratios to create complementary and synergistic products with features tailored for a particular pathophysiological condition involving skin regeneration.

## 10. Conclusions and Future Directions

Skin wound repair remains a considerable burden on healthcare systems throughout the world. While the majority of lesions heal rapidly and completely using current standard-of-care approaches, a sizable subset of patients with impaired angiogenesis and vascularization remain at high risk for the development of chronic wounds with associated co-morbidities of infection, sepsis, and osteomyelitis. Adipose tissue and blood-derived cells, exosomes, and hydrogels, as well as plant-derived cellulose, offer potential improvements in therapy. There are potential advantages and limitations associated with each modality (summarized for hydrogels in Table 2). While subcutaneous injection of autologous or allogeneic cells can modulate skin regeneration through prolonged release of paracrine factors and secretion of extracellular matrix proteins, the cell isolation, cryo-storage, and lot release criteria required for autologous cells manufactured at point of care all present logistical limitations. The ability to manufacture a secreted exosome product that is freeze-dried and stable at room temperature for extended periods is attractive; however, exosomes alone may fail to deliver the benefits provided by extracellular proteins and will require extensive evaluation before achieving regulatory approval. Complementing exosomes with adipose-, blood-, or cellulose-derived hydrogels, either as cryo-preserved injectables or lyophilized topical products, may be necessary. Whether used alone or as a combination product, hydrogels are likely to require randomized, controlled clinical trials before qualifying for regulatory approval.

## Figures and Tables

**Table 1:** Extracellular matrix-, cell-, and growth-factor-based products for the treatment of skin wounds and burns [26,27,28,29,30,31].

Type	Description	Commercial Products
Extracellular matrix (ECM)	Human acellular dermis	GRAFTJACKET^TM^ (Wright Medical Technology, Inc., Arlington, TN, USA) AlloDerm^®^ (Biohorizons implant systems Inc., Hoover, AL, USA) DermACELL AWM^®^ (LifeNet Health Inc., Virginia Beach, VA, USA) DermaMatrix^TM^ Acellular matrix (Synthes, Inc., West Chester, PA, USA) Surederm (Hans Biomed Corp., Seoul, Korea)
	Human acellular amniotic membrane	Neox^®^ (Amniox Medical, Miami, FL, USA) Epifix^®^ (MiMedx Group Inc, Marietta, GA, USA)
	Porcine acellular dermis	EZ-DERM^®^ (Mölnlycke Health Care AB, Gothenburg, Sweden),
	Porcine acellular small intestinal submucosa	OASIS^®^ Wound Matrix (Healthpoint, Fort Worth, TX, USA)
	Porcine acellular urinary bladder matrix	MicroMatrix^®^ (ACell, Columbia, MD, USA)
	Porcine dermal collagen cross-linked with fine nylon mesh	Biobrane^®^ (Smith & Nephew, London, UK)
	Bovine acellular dermis	Integra^®^ (Integra LifeSciences, Plainsboro, NJ, USA)
	Sheep fore-stomach submucosa	Endoform^®^ (Aroa Biosurgery Ltd. Auckland, New Zealand)
	Fibrin-based sealant	Tisseel^®^ (Baxter International, Deerfield, IL, USA) Evicel^®^ (Ethicon Inc., Somerville, NJ, USA)
	Hyaluronic-acid-based skin substitute	Hyaff^®^ (Fidia Advanced Biopolymers, Abano Terme, Italy)
Cells/ECM	Cultured autologous keratinocytes	Epicel^®^ (Genzyme, Cambridge, MA, USA)
	Autologous epidermal cells in liquid suspension	ReCell^®^ (Avita Medical, Cambridge, UK)
	Bovine collagen I gel seeded with neonatal foreskin fibroblasts and keratinocytes	Apligraf^®^ (Organogenesis, Canton, MA, USA)
Cells/Synthetic matrix	PLGA scaffold seeded with neonatal fibroblasts	Dermagraft^®^ (Organogenesis, Canton, MA, USA)
Growth factor	Human recombinant platelet-derived growth factor gel	Regranax^®^ (Ortho-McNeil Pharmaceutical, Raritan, NJ, USA)

**Table 2:** Hydrogel summary: physicochemical, in vitro, and in vivo properties.

Properties		AdipoGel	Cellulose	ObaGel
Physicochemical	Source	Adipose	Plant	Blood
	Enriched proteins or polymers	ECM	Cellulose	Angio/Coag
	Chemical modifiability	++	++	++
	Potential stability at room temperature	++	+++	++
In vitro	Stromal-cell-compatible	ASC	TBD	ASC
	ASC differentiation	Adipogenic and osteogenic	TBD	Adipogenic and endothelial
In vivo	Angiogenic	+	TBD	+++
	Soft tissue regeneration	++	TBD	+
	Hemostatic	TBD	++	TBD
	Antimicrobial	TBD	+++	TBD
Potential for synergy in combination		+++	+++	+++

Abbreviations: Angio, Angiogenic; ASC, Adipose Stromal/Stem Cells; Coag, Coagulation; ECM, Extracellular Matrix; TBD, To Be Determined. + = strong, ++ =stronger, +++ =strongest

## Supplementary Materials

The following are available online at https://www.mdpi.com/2218-273X/10/10/1373/s1, Sheet 1: Excel spreadsheet of Obagel vs. Matrigel proteomes sorted by relative enrichment, Sheet 2: Excel spreadsheet of Panther analysis of depleted proteins (ObaGel vs. Matrigel), Sheet 3: Excel spreadsheet of Panther analysis of enriched proteins (ObaGel vs. Matrigel).
