# Supplementary material for: Clinical Translational Potential in Skin Wound Regeneration for Adipose-Derived, Blood-Derived, and Cellulose Materials: Cells, Exosomes, and Hydrogels
Source: Biomolecules. 2020 Sep 27;10(10):1373. doi: 10.3390/biom10101373 (PMC7650547; doi:10.3390/biom10101373)
Supplement: Supplementary file 1 [file biomolecules-10-01373-s001.zip › Supplementary materials/biomolecules-937132-final-supplementary materials.pdf]

## Supplementary materials

**Supplementary Methods:** Proteomic analysis of ObaGel and Matrigel.

Quantitative proteomic analysis of ObaGel and Matrigel was performed using tandem mass tag (TMT)-based liquid chromatography–mass spectrometry (LC-MS) as described in previous studies [1]. Briefly, protein extracts from ObaGel and Matrigel were digested with trypsin (Promega Corp; Madison, WI) followed by desalting to yield lysine- and arginine-cleaved peptides. The peptides were labeled using the TMT Isobaric Mass Tagging kit (Thermo Fisher Scientific; # 90060-90061; Waltham, MA). Two aliquots (100 μg) of digested peptides obtained from ObaGel were labeled with TMT128 and 129, and two aliquots (100 μg) of peptides from Matrigel were labeled with TMT 130 and 131. The labeled peptide aliquots were then pooled together for proteomic analysis. The peptide samples were separated by an automated Easy-nLC1000 system coupled with a Q-Exactive mass spectrometer (Thermo Finnigan, USA) using a Thermo Scientific™ Acclaim™ PepMap™ 100 C18 HPLC Columns (75 μm × 150 mm, 2 μm) over a 136 min program. The identification of proteins was performed using Proteome Discoverer™ software v2.3 (Thermo-Fisher Scientific; Waltham, MA) and Mascot v2.6 (Matrix Science, Boston, MA), based on "Uniprot Human" database. The proteins that were identified on the basis of at least one unique peptide were included in the data. The ratios of TMT reporter ion abundance in ObaGel and Matrigel were used to calculate protein relative abundance.

**Supplementary Excel**

Excel spreadsheet of Obagel vs. Matrigel proteomes sorted by relative enrichment (Sheet 1)
Excel spreadsheet of Panther analysis of depleted proteins (ObaGel vs. Matrigel) (Sheet 2)
Excel spreadsheet of Panther analysis of enriched proteins (ObaGel vs. Matrigel) (Sheet 3)
